# Adult HIV care resources, management practices and patient characteristics in the Phase 1 IeDEA Central Africa cohort

**DOI:** 10.7448/IAS.15.2.17422

**Published:** 2012-11-21

**Authors:** Kimon Divaris, Jamie Newman, Jennifer Hemingway-Foday, Wilfred Akam, Ashu Balimba, Cyrille Dusengamungu, Lucien Kalenga, Marcel Mbaya, Brigitte Mfangam Molu, Veronicah Mugisha, Henri Mukumbi, Jules Mushingantahe, Denis Nash, Théodore Niyongabo, Joseph Atibu, Innocent Azinyue, Modeste Kiumbu, Godfrey Woelk

**Affiliations:** 1 Department of Pediatric Dentistry, UNC School of Dentistry, University of North Carolina-Chapel Hill, North Carolina, USA; 2 Department of Epidemiology, Gillings School of Global Public Health, University of North Carolina-Chapel Hill, North Carolina, USA; 3 RTI International, Statistics and Epidemiology, Research Triangle Park, North Carolina, USA; 4 Limbé Provincial Hôpital, Limbé, Cameroon; 5 Hôpital Militaire, Yaoundé, Cameroon; 6 Kibuye District Hospital, Kibuye, Rwanda; 7 AMO-Congo, Lubumbashi, Democratic Republic of the Congo; 8 AMO-Congo, Matadi, Democratic Republic of the Congo; 9 Hôpital Général de Yaoundé, Yaoundé, Cameroon; 10 International Center for AIDS Care and Treatment Programs, Mailman School of Public Health, Columbia University, Kigali, Rwanda; 11 AMO-Congo, Kinshasa, Democratic Republic of the Congo; 12 Muhima District Hospital, Kigali, Rwanda; 13 International Center for AIDS Care and Treatment Programs, Department of Epidemiology, Mailman School of Public Health, Columbia University, New York, NY, USA; 14 Centre Hospitalo-universitaire de Kamenge, Bujumbura, Burundi; 15 Ecole de Santé Publique, Faculté de Médecine, Kinshasa, Democratic Republic of the Congo; 16 Vindata Solutions, Yaoundé, Cameroon

**Keywords:** HIV care and treatment, Central Africa, clinical cohort, resource-limited settings

## Abstract

**Introduction:**

Despite recent advances in the management of HIV infection and increased access to treatment, prevention, care and support, the HIV/AIDS epidemic continues to be a major global health problem, with sub-Saharan Africa suffering by far the greatest humanitarian, demographic and socio-economic burden of the epidemic. Information on HIV/AIDS clinical care and established cohorts’ characteristics in the Central Africa region are sparse.

**Methods:**

A survey of clinical care resources, management practices and patient characteristics was undertaken among 12 adult HIV care sites in four countries of the International Epidemiologic Databases to Evaluate AIDS Central Africa (IeDEA-CA) Phase 1 regional network in October 2009. These facilities served predominantly urban populations and offered primary care in the Democratic Republic of Congo (DRC; six sites), secondary care in Rwanda (two sites) and tertiary care in Cameroon (three sites) and Burundi (one site).

**Results:**

Despite some variation in facility characteristics, sites reported high levels of monitoring resources, including electronic databases, as well as linkages to prevention of mother-to-child HIV transmission programs. At the time of the survey, there were 21,599 HIV-positive adults (median age=37 years) enrolled in the clinical cohort. Though two-thirds were women, few adults (6.5%) entered HIV care through prevention of mother-to-child transmission services, whereas 55% of the cohort entered care through voluntary counselling and testing. Two-thirds of patients at sites in Cameroon and DRC were in WHO Stage III and IV at baseline, whereas nearly all patients in the Rwanda facilities with clinical stage information available were in Stage I and II. WHO criteria were used for antiretroviral therapy initiation. The most common treatment regimen was stavudine/lamivudine/nevirapine (64%), followed by zidovudine/lamivudine/nevirapine (19%).

**Conclusions:**

Our findings demonstrate the feasibility of establishing large clinical cohorts of HIV-positive individuals in a relatively short amount of time in spite of challenges experienced by clinics in resource-limited settings such as those in this region. Country differences in the cohort's site and patient characteristics were noted. This information sets the stage for the development of research initiatives and additional programs to enhance adult HIV care and treatment in Central Africa.

## Introduction

Despite the recent advances in the management of HIV infection and the increased access to treatment, prevention, care and support, the HIV/AIDS epidemic continues to be a major global health problem [1]. Treatment regimens with highly active antiretroviral therapy (HAART) have transformed the infection to a manageable condition [2], and quantifiable improvements in many indicators are evident due to the HIV/AIDS programs internationally [3]. However, the epidemic confers disproportionate humanitarian, demographic and socio-economic impacts in certain areas of the world [4,5].

Sub-Saharan Africa suffers by far the greatest burden of the epidemic [4,6], and this poses extreme pressures in the affected countries’ capacities and human resources [7]. The scaling up of HAART [8,9] and voluntary HIV counselling and testing (VCT) in combination with immediate antiretroviral therapy (ART) [10], as well as care delivery and monitoring systemic improvements [11], have shown promising results. In spite of these great steps forward, enormous and diverse challenges, such as late ART initiation and high rates of loss to follow-up, still persist [12–14].

In response to the “global imperative” created by the epidemic [15,16], an unprecedented array of scientific and financial resources has been mobilized, along with a global political commitment expressed in the Millennium Development Goals [17]. Long-lasting scientific initiatives [18,19] in industrialized nations have advanced the current state of knowledge and care of those with HIV infection and AIDS. During the last decade, increased funding allocation was made possible via major funders such as the US President's Emergency Plan for AIDS Relief (PEPFAR), The Global Fund to Fight AIDS, Tuberculosis, and Malaria, and the World Bank. These resources provided new opportunities for HIV/AIDS prevention, treatment and care in low-resource regions [20].

This scaling-up created an immediate need for high-quality research and implementation science to help inform the scale-up process, as well as effective planning, monitoring and evaluation of HIV/AIDS related programs, through international collaborations [21,22]. In response, the International Epidemiologic Databases to Evaluate AIDS (IeDEA) was initiated in 2006 with funding from the US National Institutes of Health (NIH), and the National Institute of Allergy and Infectious Diseases (NIAID) to function as a data harmonization and collaboration platform. The Eunice Kennedy Shriver National Institute of Child Health and Development (NICHD) provides additional support to conduct paediatric research, and the National Cancer Institute (NCI) to initiate cancer-related research in Africa through the IeDEA regional centres and affiliated sites. Currently, there are seven funded IeDEA participating regions in five continents. The present paper describes the results of a survey that was undertaken among the adult HIV care facilities of the IeDEA-Central Africa (CA) Phase 1 network with the goals of summarizing, comparing and presenting information on clinical care resources, management practices and patient characteristics within the regional network.

## Methods

### The IeDEA-CA network

The international research consortium of IeDEA (www.idea.org) has established regional centres that monitor high-quality data collection, entry and storage, in a uniform or compatible manner across the seven regions: North America [23], Latin America and Caribbean [24], Asia/Pacific, West Africa, Central Africa, East Africa and Southern Africa. IeDEA enables researchers around the world to address novel and evolving research questions in HIV/AIDS that are currently unanswerable by single cohorts. A description of the entire IeDEA sub-Saharan Africa clinical cohort profile has been recently published [25]. From 2006 to 2011, the Phase 1 Central Africa collaboration, in particular, consisted of a total of 13 facilities providing adult HIV care and treatment (10 adult HIV care and treatment clinics from Burundi, Cameroon, and Democratic Republic of Congo (DRC) plus three adult clinics in Rwanda) and four facilities providing paediatric HIV care and treatment in Burundi, Cameroon and DRC. Country-level information on population characteristics and prevalence estimates of HIV infection and ART for these four countries based on the UNAIDS 2008 Report on the Global AIDS epidemic [26–29] are presented in Table 1.

**Table 1:** Country population profile and HIV prevalence and treatment characteristics

	Burundi	Cameroon	DR Congo	Rwanda
Demographic characteristics				
Population (thousands)	8508	18,549	62,636	9725
% of population urban	11	56	33	21
Gross national income per capita ($) (in 2006)	320	2060	270	730
HIV infection and treatment characteristics				
Estimated adult HIV prevalence (95% CL)^a^	2.0 (1.3, 2.5)	5.1 (3.9, 6.2)	1.2, 1.5	2.8 (2.4, 3.2)
Estimated antiretroviral therapy among clinically eligible adults (95% CL)	23 (18, 31)	25 (21, 32)	20, 29	71 (62, 84)

Source: UNAIDS 2008 Country HIV/AIDS Epidemiological Profiles.

CL, confidence limits.

a ‘Low’ and ‘high’ estimates only reported for DRC.

Data were collected and entered at participating health facilities into separate paediatric and adult databases and merged at the data coordinating centre at RTI International in the United States. The collaboration built on the existing partnerships between the Kinshasa School of Public Health, RTI International, the University of North Carolina at Chapel Hill Gillings School of Global Public Health, as well as the Institute of Tropical Medicine Antwerp, Belgium. Local collaborators included the HIV/AIDS clinicians/care providers, representatives of national HIV/AIDS programs, faculty from medical schools in the region, as well as the International Center for AIDS Care and Treatment Programs (ICAP) at Columbia University that contributed data from Rwanda.

### The IeDEA-CA clinical cohort

The IeDEA-CA Phase 1 clinical cohort grew from 800 HIV-positive individuals in 2007 to approximately 22,000 by the end of 2010: 21,599 adults and 425 children who were diagnosed as HIV positive. For the purposes of this study, we focused on the clinics providing adult care and did not include four participating paediatric care centres, one in DRC, two in Cameroon and one in Burundi, due to differences in paediatric and adult cohort characteristics. We also excluded one site with exclusive care of female patients in Rwanda because this site differed from the other sites in the IeDEA-CA consortium in terms of the unique population that it serves (the majority are survivors of genocidal rape).

### Data collection and analysis

Periodic assessments to obtain information on site resources, facilities, clinical practices, staffing, patient attendance and care, and additional services or programs offered at the site were undertaken within the IeDEA-CA collaboration. For the present study, we used responses from a Web-based survey administered in October 2009 to collect data on (1) site characteristics including level of care (primary, secondary and tertiary), staffing characteristics and storage of medical records; (2) care and treatment characteristics including points of entry into care and costs incurred by patients for services; and (3) program characteristics including availability of clinical and support services, ART availability, patient follow-up and the diagnosis and management of HIV-associated illnesses. The survey could be completed directly online or, for sites with limited internet access, printed and then entered into the Web-based system. All 12 facilities included in this analysis completed the survey.

In terms of patient characteristics, sites in the IeDEA-CA network collected standardized baseline data for each participant, as well as follow-up data at subsequent visits. Patients were followed at regular intervals dictated by their ongoing care at each local facility. More specifically, patients on ART were followed every one to three months, and those not on ART were followed every six months, unless there was a clinical event for which they needed to return to the clinic for evaluation and/or care. A facility doctor or nurse attended all patients. The same data collection forms were used at participating facilities in Burundi, Cameroon and DRC to collect data on socio-demographics, anthropometrics, risk behaviours, medical diagnoses (co-morbidities), laboratory values (CD4 counts, viral load, etc.), medications (antiretroviral (ARV), cotrimoxazole prophylaxis, TB prophylaxis and treatment) and dates of initiation, referrals and outcomes such as vital status and loss to follow-up. Data were abstracted from the existing electronic database at the participating sites in Rwanda and harmonized with the IeDEA-CA database. Data analysis relied predominantly upon descriptive methods and summary estimates. To enable comparisons between male and female participants, sex-stratified patient characteristics are also presented. To facilitate interpretation of these differences, *p* values derived from tests of comparison of the stratified estimates (χ^2^ test for categorical variables; Student's *t* or median tests for continuous variables) are presented. The statistical program Stata 12.1 (StataCorp LP, College Station, TX) was used for all analyses.

### Human rights protection

The IeDEA-CA region research project was reviewed by the Institutional Review Board (IRB) at the Kinshasa School of Public Health in DRC, the national ethics committees in Burundi and Cameroon and the data coordinating centre IRB at RTI International. IRB approval was also obtained from the Rwanda National Ethics Committee to abstract data from already existing databases managed by the participating hospitals and that were made available to the International Center for AIDS Care and Treatment Programs (ICAP) at Columbia University and the Rwanda Women's Inter-Association Study (RWISA).

## Results

### Site and program characteristics

The 12 participating adult care sites were a combination of hospital and ambulatory care units of varying size/capacity, serving predominantly urban populations, and offering primary care in DRC, secondary care in Rwanda and tertiary care in Burundi and Cameroon (Table 2). All sites maintained on-site electronic databases for data storage and had a unique identifier for every patient. All sites recorded patients’ education level and socio-economic status. While internet availability varied, regular transmissions ensured that the data coordinating centre database was up-to-date with the local databases at participating facilities. All sites documented drug stock-outs and reported some disruption of ARV medications during the last year, with duration that varied from one day in Rwanda to more than three weeks in DRC and Cameroon. All sites indicated that there were disruptions in prophylactic or opportunistic infection drug supply in the past year. Provision of routine HIV testing for patients’ relatives, sex partners and household members was available in all sites, while some level of linkage to prevention of mother-to-child transmission (PMTCT) of HIV programs was also reported. All sites recorded history of PMTCT for female patients. With the exception of Burundi, the programs offered nutritional counselling to their patients and performed frequent anthropometric evaluations. A summary of program characteristics is presented by country in Table 2.

**Table 2:** The IeDEA-CA clinical care resources and management practices

	Program sites in Burundi	Program sites in Cameroon	Program sites in DR Congo	Program sites in Rwanda
**Electronic infrastructure and documentation**				
Availability of internet for the support staff (percent of time)	80	100	0^b^	100
Availability of internet for the clinical staff (percent of time)	50	50^a^	0^b^	100^e^
Record a national IDF or government ID for every patient	No	No^a^	No^c^	Yes
Possible to link a patient's records to a child's records	No	No	Yes^d^	Yes^e^
Possible to link a patient's records to a spouse or partner's records	No	No	No^b^	Yes^e^
What CD4 criteria are used for ART initiation at each WHO stage?				
WHO 1	<350	<350	<200	<350
WHO 2	<350	<350	<200	<350
WHO 3	Any	<200	<350	<350
WHO 4	Any	Any	Any	Any
**Monitoring and availability of drugs/services**				
Disruptions of ARV drug supply in the past year	Yes	Yes	Yes	No
How long was the worst disruption in ARV drug supply in the last six months?	Two to seven days	>21 days	>21 days	One day
Relationship of HIV with PMTCT programs	Both on-site and linked	Both on-site and linked^a^	PMTCT program is off-site and linked to care and treatment program^c^	Both on-site and linked
Provision of nutritional counseling	No	Yes	Yes^c^	Yes
Frequency of anthropometric evaluation for adult patients	Not done	Once every three months	Every one to three months	Once a month
**Costs incurred by patients for services**				
Screening consult	No (free)	Full pay^a^	No (free)	No (free)
Laboratory tests	No (free)	Partial pay^a^	Partial pay^d^	No (free)
Diagnostic exams	Partial pay	Full pay	No (free)^b^	No (free)^e^
First-line ART	No (free)	No (free)	No (free)	No (free)
Second-line ART	No (free)	No (free)	No (free)	No (free)
OI prophylaxis	Partial pay	No (free)^a^	No (free)^b^	No (free)
OI treatment	Partial pay	Full pay	No (free)^b^	No (free)^e^
Routine follow-up consult	No (free)	Full pay^a^	No (free)	No (free)
Viral load tests	No (free)	Full pay	Full pay	No (free)

ART, antiretroviral therapy; ARV, antiretroviral drugs; IeDEA-CA, International Epidemiologic Databases to Evaluate AIDS Central Africa; IDF/ID, identification document; OI, opportunistic infection; PMTCT, prevention of mother-to-child transmission of HIV; WHO, World Health Organization.

a Denotes in 2/3 sites in Cameroon

b 5/6 sites

c 4/6 sites

d in 3/6 sites in DR Congo

e in 1/2 sites in Rwanda.

### Patient characteristics

The IeDEA-CA adult cohort characteristics are presented in Table 3. DRC had 9871 patients, followed by Cameroon (*n=*4922), Rwanda (*n* **=**4583) and Burundi (*n=*2223). Patients’ mean (median) age ranged from 34 (33) years in Rwanda to 40 (40) in DRC. Marked differences were evident in disease status and mean CD4 counts between Rwanda and DRC, although these estimates were based on small sub-sets of patients that had CD4 values available. In DRC and Cameroon, two-thirds of patients were in WHO clinical Stage III or IV at enrolment into the IeDEA-CA database and virtually all (97%) of the 3224 patients with clinical stage information available in the Rwandan sites were in Stage I or II. One-third of participants in the Rwanda site had missing clinical stage information, whereas very few of the participants at the sites in the other three countries had missing clinical stage. More than half of the patients at the sites in DRC and Rwanda and 80% of the patients at the Burundi sites entered HIV care through VCT. About two-thirds of patients enrolled in each country were women though very few entered HIV care through PMTCT services in Burundi (3%), Cameroon (4%) and DRC (0.6%). About one-quarter of the patients in Rwanda entered HIV care through PMTCT.

**Table 3:** The IeDEA-CA adult clinical cohort characteristics by country, overall and stratified by sex

	Program sites in Burundi				Program sites in Cameroon				Program sites in DR Congo				Program sites in Rwanda			

Number of sites	1				3				6				2			
Number of adult patients (*n*)	2223				4922				9871				4583			
Sex																
Female, *n* (%)^a^	1536 (69)				3437 (70)				6726 (68)				3095 (68)			
Male, *n* (%)^a^	686 (31)				1482 (30)				3096 (32)				1488 (32)			
Missing, *n*	1				3				49				0			
				
	All (%)^a^	Female (%)	Male (%)	*p* b	All (%)^a^	Female (%)	Male (%)	*p* b	All (%)^a^	Female (%)	Male (%)	*p* b	All (%)^a^	Female (%)	Male (%)	*p* b
Socio-demographics																
Age (years; mean)	38	36	42	<0.05^c^	38	37	41	<0.05^c^	40	38	43	<0.05^c^	34	32	38	<0.05^c^
Marital status																
Married	35	28	52		33	24	53		42	32	64		30	25	41	
Single/living together but not married	32	33	31	<0.05	47	51	36	<0.05	23	25	19	<0.05	53	54	51	<0.05
Divorced	9	11	7		5	6	3		11	13	7		2	2	1	
Widowed	23	28	11		16	19	7		24	31	11		15	19	7	
Missing (*n*)	1	1	0		0	0	0		415	296	96		1876	1315	561	
Perceived discordancy with partner																
Yes	7	6	10	<0.05	20	17	26	<0.05	8	7	11	<0.05	30	27	38	<0.05
No	35	29	50		23	20	30		23	21	28		13	10	17	
Do not know	16	18	12		32	33	29		46	47	45		12	14	7	
N/A or missing	41	46	28		25	30	15		23	26	16		45	49	38	
Pregnant																
Yes		4				4				3				15		
No		96				96				97				85		
Missing (*n*)		2				0				47				0		
Clinical characteristics																
WHO Clinical Stage																
I	33	33	33	0.10	16	17	12	<0.05	9	10	8	0.09	64	67	59	<0.05
II	17	17	16		19	20	18		24	24	24		33	31	36	
III	36	37	34		50	50	52		61	61	61		3	2	5	
IV	14	13	17		15	13	18		6	6	6		0	0	0	
Missing (*n*)	7	4	2		17	8	6		125	73	31		1359	830	529	
CD4 count (median)		318	253	<0.05^d^		219	182	<0.05^d^		235	187	<0.05^d^		440	306	<0.05^d^
missing (*n*)		700	323		861	611	248		8009	5418	2556		3823	2560	1263	
ART																
Initiated prior to enrolment	50	52	43	<0.05	49	49	47	0.62	27	28	27	0.57	0.1	0.2	0	0.19
Initiated at enrolment	5	5	7		36	36	38		2	1	2		6	5	6	
Stopped at enrolment	1	0.8	0.3		0.1	0.1	0.1		0.4	0.4	0.4		0	0	0	
Never (ART-naïve)	44	42	50		15	15	15		71	70	71		94	94	94	
Missing (*n*)	8	6	1		18	9	6		133	75	35		0	0	0	
Entry to HIV care																
VCT	81	79	84	<0.05	40	39	42	<0.05	56	56	57	<0.05	55	50	67	<0.05
PMCTC	3	4	0		4	6	0.3		0.6	0.8	0.2		24	31	9	
TB/STI clinic or other	14	14	13		32	30	35		10	10	9		21	19	24	
No previous care	3	3	3		25	25	23		33	33	33		0	0	0	
Missing (*n*)	71	50	21		3	0	0		93	46	21		0	0	0	
TB therapy																
Yes	4	3	7	<0.05	5	4	6	<0.05	5	5	6	<0.05	0.1	0.1	0.1	0.97
No	96	97	93		95	96	94		95	95	94		100	100	100	
Missing (*n*)	8	6	1		8	3	2		124	68	28		0	0	0	

ART, antiretroviral therapy; IeDEA-CA, International Epidemiologic Databases to evaluate AIDS Central Africa; IQR, interquartile range; PMTCT, prevention of mother-to-child transmission of HIV; SD, standard deviation; STI, sexually transmitted infection; TB, tuberculosis; VCT, voluntary counseling and testing for HIV prevention; WHO, World Health Organization.

a Percentages calculated among the non-missing responses

b *p* values derived from χ^2^ tests of equivalence

c Student's *t*-test of equality of means

d test of equality of medians.

With regard to differences between male and female participants, women were consistently younger (four to eight years mean difference) than men, in all country programs. Significantly higher proportions of men were married (i.e. 64% vs. 32% in DRC), while higher proportions of women were widowed (i.e. 31% vs. 11% in DRC). Women were enrolled into the IeDEA-CA database at an earlier disease stage than men, but this difference was most pronounced in the Cameroon sites. A larger proportion of men entered HIV care through VCT as compared to women.

### Treatment regimens

Participating sites in Cameroon were the first within the IeDEA-CA region to begin offering ART for adults in 2000, while Rwandan sites started in 2004–05, and participating sites in Burundi and DRC between 2006 and 2007 (Table 2). In the IeDEA participating sites in Burundi and Cameroon, approximately half of the patients who were enrolled in HIV care had initiated ART, when the IeDEA-CA baseline form was completed, whereas this proportion was one out of four in the IeDEA sites from DRC. In Rwanda, a small proportion of patients started ART at the enrolment visit, as this facility represents a point of entry to HIV care. Although the participating IeDEA-CA facilities in the other three countries also represent a point of entry into HIV care, many of these patients had already been receiving care at these facilities when the IeDEA-CA database was rolled out. The most common treatment regimen was d4T-30+3TC+NVP (stavudine/lamivudine/nevirapine−64%), followed by AZT+3TC+NVP (zidovudine/lamivudine/nevirapine−19%). Approximately one-third of patients had a drug substitution in Cameroon, whereas this proportion was smaller in Burundi (25%) and in DRC (15%). Four percent of patients underwent a change from first to second line regimens in Cameroon and Burundi, and less than 1% in DRC.

## Discussion

The unique challenges of HIV care in sub-Saharan Africa require a “systems approach” based on operational research and a solid evidence-base [30,31]. Gaining a pragmatic and comprehensive understanding of the current HIV care characteristics in the region is a pre-requisite for any intervention or “fine-turning” of current practices, re-allocation of resources or other programmatic changes. The data presented here provide insights into HIV care within the Central Africa region and can set the stage for additional hypothesis-generating research and subsequent longitudinal investigations. It is imperative, however, that these observations are considered in the context of regional and country-level framing factors, to gain understanding into possible program-implementation gaps and differences [32].

### Between-country and -site differences: structural barriers to ART

Country-level differences that frame HIV care are evident in the domains of urban/rural population ratio (e.g. Burundi: 11% vs. Cameroon: 56%), the estimated prevalence of HIV infection (DRC: 1.2–1.5% vs. Cameroon: 5.1%) and the proportion of eligible adults receiving ART (approximately 20% for all countries except Rwanda: 71%) [26–29]. Serious challenges have been reported in the implementation phase of ART programs in the region, and among those are important structural, health system, social and behavioural barriers to ART initiation and adherence [33,34]. According to recent reports, the availability and adequacy of human resources in the health sector has been a major ART implementation impediment in Burundi [35], whereas decentralization, HIV testing and referrals, and ARV supply have been additional structural barriers in Cameroon [36–38]. Conversely, Rwanda has often been regarded as an example of successful build-up of health system infrastructures and decentralization of services across the country [39].

Studies based on quantitative [40] and qualitative [41,42] methodology have provided valuable insights into the multi-level factors affecting patients’ access to, initiation, and adherence to ART in the region. “Common themes” include patients’ difficulty accessing HIV care sites due to distance, work and long waiting lines [41,43,44], health personnel number and training inadequacies [45], infrastructure insufficiencies [46], poverty [47], migration [48] and others. These factors (e.g. distance to the clinic) are important parameters when one considers the catchment population of the participating facilities, which served exclusively urban populations in Rwanda and DRC (with the exception of one site) and both urban and rural in Burundi and Cameroon. They also need to be taken into consideration when comparing the demographic and clinical profile of each country's cohort (i.e. compared to patients in the DRC sites, those followed in the Rwandan facilities had a seven-year younger median age and most were in WHO Stage I vs. III in DRC). Noteworthy, the large differences that were observed in ART initiation status and CD4 counts were affected by country characteristics, but also by each site's reference population (rural/urban), entry to HIV care and more.

Noteworthy, all sites used the then-current WHO criteria for ART initiation and treatment was offered free of charge to patients. Of course, entry to HIV care is of paramount importance, as recent findings among HIV-infected patients in sub-Saharan Africa [49] indicate that much of the excess mortality experienced among seropositive patients might be prevented by more timely initiation of ART.

Rwanda is the only country where participating IeDEA sites reported no ARV drug stock-outs and supply interruptions in the past year. This is an important finding, as hospital pharmacy ARV shortage was identified as a significant predictor of ART non-adherence among a sample of Cameroonian patients, in Yaoundé [40]. As mentioned above, patients enrolled in Rwanda were younger, tended to be enrolled into care at WHO clinical Stage I or II and had, acknowledging the missing data issue, higher CD4 counts. This observation, although limited by the non-representativeness of the study sample, is consistent with the high ART-enrolment proportion in Rwanda – a PEPFAR-focus country, and the only focus country among our Central Africa cohort. Rwanda is one of eight low- and middle-income countries (and one of only two in Africa) that had achieved universal access to ART by December 2009 [50]. In 2008, PEPFAR was re-authorized with funds of nearly $50 billion and included a focus on strengthening existing health systems [20,51].

### By-sex differences

Because sex-related disparities in HIV care are commonly reported [52,53], the presentation of sex-stratified patient characteristics of the IeDEA-CA clinical cohort is of interest. For example, recent data among HIV-infected individuals under care showed that women may experience higher mortality than men, even after controlling for HAART utilization [54]. In the current study, a larger proportion of men presented with Stage IV disease in sites in Cameroon and Burundi and had lower CD4 counts in all sites, which is consistent with other recent findings [55]. This, along with the observed demographic differences (men were older and more likely to be married), can be attributed to men's delay in seeking HIV care. Similarly, men's higher access via VCT may be due to their experience of disease symptoms, or self-definition as being at risk for HIV infection. More research is required to disentangle the contribution of social, cultural, behavioural, or biological and immunological factors [56–59] to these by-sex differences.

### Strengths and limitations

The main strength of the IeDEA-CA is the cohort's large number of seropositive individuals for whom socio-demographic and anthropometric data, clinical values, co-morbid conditions and treatment status were available. Moreover, the participating facilities benefited from the harmonization efforts in data collection, the quality assurance procedures and the improved data collection and management. As an example, many of the participating sites in Burundi, Cameroon and DRC began to use the IeDEA-CA data collection forms as the medical charts for patient care and the accompanying database as the electronic medical record system [60]. A reporting tool, available to each facility completing the IeDEA-CA forms, enabled physicians to produce reports by key variables (i.e. gender and ARV regimens) which were useful for clinic management, fulfilling reporting requirements for funding agencies and producing enrolment graphs for presentations and other advocacy activities. A listing of study ID numbers can be produced for patients that have not returned to clinic for scheduled appointments assisting clinic staff with lost to follow-up tracking. Prior to the IeDEA-CA database and accompanying reporting tool, many physicians and staff at the participating health facilities in Cameroon, Burundi and DRC were spending large amounts of time tallying these reports manually, which took away from patient care. However, patient management issues such as loss to follow-up remain a priority area for improvement.

While the IeDEA-CA adult HIV clinical care characteristics presented in this article reflect the participating facilities’ characteristics and patients’ profile, these data are neither nationally representative nor derived from randomly selected HIV care facilities; therefore, they lack the external validity that would permit between-country comparisons and inferences. Data completeness is another major limitation. For example, there were considerable proportions of missing information for marital status in DRC and Rwanda. Moreover, we have substantial proportions of missing information on patients’ clinical stage, mainly in Rwanda, and CD4 counts, mainly in DRC, but also in Burundi and Rwanda. The extent of missing data is a reflection of systematic between-site differences, including enrolled patients’ characteristics and stage of disease/treatment; however, disentangling these influences is not possible from these data. Additionally, the cohort faces the threats that all open clinical cohorts encounter, in terms of patient retention. The problem of substantial number of patients on ART being lost to follow-up in resource-limited settings has been well-documented, and measures to maximize ART program retention are warranted [61]. All participating IeDEA-CA sites had protocols for contacting and documenting the outcomes of those lost to follow-up.

Although, according to data of the last decade, small proportions of HIV-infected individuals actually received treatment in the Central Africa region, ranging from 2% to 26%, the more than 20,000 HIV-positive individuals that are followed in IeDEA-CA network facilities represent a considerable cohort. Nevertheless, more efforts are needed in the region to step-up efforts to provide treatment to all individuals that are in need of it.

## Conclusions

The information presented in this paper outlines and provides insight into the program, site and patient characteristics with regard to HIV adult clinical care in selected facilities in Burundi, Cameroon, DRC and Rwanda. This robust cohort of more than 22,000 seropositive individuals demonstrates the feasibility of establishing large clinical cohorts of HIV-positive individuals in a relatively short amount of time in spite of challenges experienced by clinics in resource-limited settings such as those in this region. Within its limitations, the IeDEA-CA network provides a unique framework for data collection harmonization and quality assurance, while offering a plethora of research opportunities that facilitate collaborative initiatives in the region. It is envisioned that the development of future research initiatives and additional program activities will enhance adult care HIV treatment in Central Africa and beyond.

## Abbreviations

ART, antiretroviral therapy; ARV, antiretroviral; CA, Central Africa; DRC, Democratic Republic of Congo; HAART, highly active antiretroviral therapy; IeDEA, International Epidemiologic Databases to Evaluate AIDS; PMTCT, prevention of mother-to-child transmission; VCT, voluntary HIV counselling and testing.

## References

[1] UNAIDS [Internet] Joint action for results: UNAIDS outcome framework 2009–2011. http://data.unaids.org/pub/BaseDocument/2010/jc1713_joint_action_en.pdf.

[2] Hammer SM, Eron JJ, Reiss P, Schooley RT, Thompson MA, Walmsley S (2008). Antiretroviral treatment of adult HIV infection: 2008 recommendations of the International AIDS Society-USA panel. JAMA.

[3] Mahy M, Warner-Smith M, Stanecki KA, Ghys PD (2009). Measuring the impact of the global response to the AIDS epidemic: challenges and future directions. J Acquir Immune Defic Syndr.

[4] UNAIDS [Internet] AIDS epidemic. http://data.unaids.org/pub/Report/2009/2009_epidemic_update_en.pdf.

[5] Braitstein P, Brinkhof MW, Dabis F, Schechter M, Boulle A, Miotti P (2006). Mortality of HIV-1-infected patients in the first year of antiretroviral therapy: comparison between low-income and high-income countries. Lancet.

[6] Zachariah R, Ford N, Philips M, Lynch S, Massaquoi M, Janssens V (2009). Task shifting in HIV/AIDS: opportunities, challenges and proposed actions for sub-Saharan Africa. Trans R Soc Trop Med Hyg.

[7] Asamoah-Odei E, Garcia Calleja JM, Boerma JT (2004). HIV prevalence and trends in sub-Saharan Africa: no decline and large subregional differences. Lancet.

[8] Egger M, Boulle A (2008). Population effect of scaling up ART in resource-poor settings. Lancet.

[9] Gilks CF, Crowley S, Ekpini R, Gove S, Perriens J, Souteyrand Y (2006). The WHO public-health approach to antiretroviral treatment against HIV in resource-limited settings. Lancet.

[10] Granich RM, Gilks CF, Dye C, De Cock KM, Williams BG (2009). Universal voluntary HIV testing with immediate antiretroviral therapy as a strategy for elimination of HIV transmission: a mathematical model. Lancet.

[11] Zachariah R, Harries AD, Philips M, Arnould L, Sabapathy K, O'Brien DP (2010). Antiretroviral therapy for HIV prevention: many concerns and challenges, but are there ways forward in sub-Saharan Africa?. Trans R Soc Trop Med Hyg.

[12] Boyer S, Eboko F, Camara M, Abé C, Nguini ME, Koulla-Shiro S (2010). Scaling up access to antiretroviral treatment for HIV infection: the impact of decentralization of healthcare delivery in Cameroon. AIDS.

[13] Walsh A, Ndubani P, Simbaya J, Dicker P, Brugha R (2010). Task sharing in Zambia: HIV service scale-up compounds the human resource crisis. BMC Health Serv Res.

[14] van Damme W, Kober K, Laga M (2006). The real challenges for scaling up ART in sub Saharan Africa. AIDS.

[15] Collins C, Coates TJ, Szekeres G (2008). Accountability in the global response to HIV: measuring progress, driving change. AIDS.

[16] Piot P, Bartos M, Ghys PD, Walker N, Schwartländer B (2001). The global impact of HIV/AIDS. Nature.

[17] United Nations [Internet] Millennium development goals. http://www.un.org/millenniumgoals/aids.shtml.

[18] Schoeni-Affolter F, Ledergerber B, Rickenbach M, Rudin C, Günthard HF, Swiss HIV Cohort Study (2010). Cohort profile: the Swiss HIV Cohort study. Int J Epidemiol.

[19] Kaslow RA, Ostrow DG, Detels R, Phair JP, Polk BF, Rinaldo CR (1987). The multicenter AIDS cohort study: rationale, organization, and selected characteristics of the participants. Am J Epidemiol.

[20] Bendavid E, Bhattacharya J (2009). The President's emergency plan for AIDS relief in Africa: an evaluation of outcomes. Ann Intern Med.

[21] Bennett S, Boerma JT, Brugha R (2006). Scaling up HIV/AIDS evaluation. Lancet.

[22] Boerma JT, Stanecki KA, Newell ML, Luo C, Beusenberg M, Garnett GP (2006). Monitoring the scale-up of antiretroviral therapy programmes: methods to estimate coverage. Bull World Health Organ.

[23] Gange SJ, Kitahata MM, Saag MS, Bangsberg DR, Bosch RJ, Brooks JT (2007). Cohort profile: the North American AIDS Cohort Collaboration on Research and Design (NA-ACCORD). Int J Epidemiol.

[24] McGowan CC, Cahn P, Gotuzzo E, Padgett D, Pape JW, Wolff M (2007). Cohort profile: Caribbean, Central and South America Network for HIV research (CCASAnet) collaboration within the International Epidemiologic Databases to Evaluate AIDS (IeDEA) programme. Int J Epidemiol.

[25] Egger M, Ekouevi DK, Williams C, Lyamuya RE, Mukumbi H, Braitstein P (2012). Cohort profile: the international epidemiological databases to evaluate AIDS (IeDEA) in sub-Saharan Africa. Int J Epidemiol.

[26] UNAIDS/WHO Working Group on Global HIV/AIDS and STI Surveillance [Internet] Epidemiological fact sheet on HIV and AIDS. Core data on epidemiology and response.

[27] UNAIDS/WHO Working Group on Global HIV/AIDS and STI Surveillance [Internet] Epidemiological fact sheet on HIV and AIDS. Core data on epidemiology and response. http://apps.who.int/globalatlas/predefinedReports/EFS2008/full/EFS2008_CM.pdf.

[28] UNAIDS/WHO Working Group on Global HIV/AIDS and STI Surveillance [Internet] Epidemiological fact sheet on HIV and AIDS. Core data on epidemiology and response.

[29] UNAIDS/WHO Working Group on Global HIV/AIDS and STI Surveillance [Internet] Epidemiological fact sheet on HIV and AIDS. Core data on epidemiology and response.

[30] Heidari S, Harries AD, Zachariah R (2011). Facing up to programmatic challenges created by the HIV/AIDS epidemic in sub-Saharan Africa. J Int AIDS Soc.

[31] Zachariah R, Van Damme W, Arendt V, Schmit JC, Harries AD (2011). The HIV/AIDS epidemic in sub-Saharan Africa: thinking ahead on programmatic tasks and related operational research. J Int AIDS Soc.

[32] Hirschhorn LR, Ojikutu B, Rodriguez W (2007). Research for change: using implementation research to strengthen HIV care and treatment scale-up in resource-limited settings. J Infect Dis.

[33] Dong K, Thabethe Z, Hurtado R, Sibaya T, Dlwati H, Walker B (2007). Challenges to the success of HIV and tuberculosis care and treatment in the public health sector in South Africa. J Infect Dis.

[34] Bartlett JA, Hornberger J, Shewade A, Bhor M, Rajagopalan R (2009). Obstacles and proposed solutions to effective antiretroviral therapy in resource-limited settings. J Int Assoc Physicians AIDS Care (Chic).

[35] Cailhol J, Mathole T, Parsons A, Sanders D, Kandondo D, Ndayiragije I (2009). Burundi: building a health system together with global health initiatives, in the aftermath of war. The maximizing positive synergies academic consortium: interactions between global health initiatives and health systems: evidence from countries [Internet].

[36] Boyer S, Eboko F, Camara M, Abe C, Owona Nguini ME, Koulla-Shiro S (2009). Cameroon: evaluation of the national programme for access to antiretroviral therapy. The maximizing positive synergies academic consortium: interactions between global health initiatives and health systems: evidence from countries [Internet].

[37] Loubiere S, Boyer S, Protopopescu C, Bonono CR, Abega SC, Spire B (2009). Decentralization of HIV care in Cameroon: increased access to antiretroviral treatment and associated persistent barriers. Health Policy.

[38] Boyer S, Eboko F, Camara M, Abé C, Nguini ME, Koulla-Shiro S (2010). Scaling up access to antiretroviral treatment for HIV infection: the impact of decentralization of healthcare delivery in Cameroon. AIDS.

[39] Mukherjee JS, Jerome JG, Sullivan E, May MA, Mayfield A, Lambert W (2009). Rwanda: the impact of global health initiatives on the health system: a mixed methods analysis. The maximizing positive synergies academic consortium: interactions between global health initiatives and health systems: evidence from countries [Internet].

[40] Marcellin F, Boyer S, Protopopescu C, Dia A, Ongolo-Zogo P, Koulla-Shiro S (2008). Determinants of unplanned antiretroviral treatment interruptions among people living with HIV in Yaoundé, Cameroon (EVAL survey, ANRS 12-116). Trop Med Int Health.

[41] Dahab M, Charalambous S, Hamilton R, Fielding K, Kielmann K, Churchyard GJ (2008). “That is why I stopped the ART”: patients’ & providers’ perspectives on barriers to and enablers of HIV treatment adherence in a South African workplace programme. BMC Public Health.

[42] Roura M, Busza J, Wringe A, Mbata D, Urassa M, Zaba B (2009). Barriers to sustaining antiretroviral treatment in Kisesa, Tanzania: a follow-up study to understand attrition from the antiretroviral program. AIDS Patient Care STDS.

[43] Mshana GH, Wamoyi J, Busza J, Zaba B, Changalucha J, Kaluvya S (2006). Barriers to accessing antiretroviral therapy in Kisesa, Tanzania: a qualitative study of early rural referrals to the national program. AIDS Patient Care STDS.

[44] Fredlund VG, Nash J (2007). How far should they walk? Increasing antiretroviral therapy access in a rural community in northern KwaZulu-Natal, South Africa. J Infect Dis.

[45] Martinot A, Van Rie A, Mulangu S, Mbulula M, Jarrett N, Behets F (2008). Baseline assessment of collaborative tuberculosis/HIV activities in Kinshasa, the Democratic Republic of Congo. Trop Doct.

[46] Petti CA, Polage CR, Quinn TC, Ronald AR, Sande MA (2006). Laboratory medicine in Africa: a barrier to effective health care. Clin Infect Dis.

[47] Boyer S, Marcellin F, Ongolo-Zogo P, Abega SC, Nantchouang R, Spire B (2009). Financial barriers to HIV treatment in Yaoundé, Cameroon: first results of a national cross-sectional survey. Bull World Health Organ.

[48] Kagee A, Remien RH, Berkman A, Hoffman S, Campos L, Swartz L (2011). Structural barriers to ART adherence in Southern Africa: challenges and potential ways forward. Glob Public Health.

[49] Brinkhof MW, Boulle A, Weigel R, Messou E, Mathers C, Orrell C (2009). Mortality of HIV-infected patients starting antiretroviral therapy in sub-Saharan Africa: comparison with HIV-unrelated mortality. PLoS Med.

[50] WHO/UNAIDS/UNICEF [Internet] (2010). Towards universal access: scaling up priority HIV/AIDS interventions in the health sector. Progress Report.

[51] El-Sadr WM, Hoos D (2008). The President's Emergency Plan for AIDS Relief–is the emergency over?. N Engl J Med.

[52] Abdool Karim SS, Churchyard GJ, Abdool Karim Q, Lawn SD (2009). HIV infection and tuberculosis in South Africa: an urgent need to escalate the public health response. Lancet.

[53] McNaghten AD, Hanson DL, Dworkin MS, Jones JL (2003). Adult/Adolescent Spectrum of HIV Disease Group. Differences in prescription of antiretroviral therapy in a large cohort of HIV-infected patients. J Acquir Immune Defic Syndr.

[54] Lemly DC, Shepherd BE, Hulgan T, Rebeiro P, Stinnette S, Blackwell RB (2009). Race and sex differences in antiretroviral therapy use and mortality among HIV-infected persons in care. J Infect Dis.

[55] Hawkins C, Chalamilla G, Okuma J, Spiegelman D, Hertzmark E, Aris E (2011). Sex differences in antiretroviral treatment outcomes among HIV-infected adults in an urban Tanzanian setting. AIDS.

[56] Nicastri E, Leone S, Angeletti C, Palmisano L, Sarmati L, Chiesi A (2007). Sex issues in HIV-1-infected persons during highly active antiretroviral therapy: a systematic review. J Antimicrob Chemother.

[57] Marcellin F, Abé C, Loubière S, Boyer S, Blanche J, Koulla-Shiro S (2009). Delayed first consultation after diagnosis of HIV infection in Cameroon. AIDS.

[58] Mavhu W, Dauya E, Bandason T, Munyati S, Cowan FM, Hart G (2010). Chronic cough and its association with TB-HIV co-infection: factors affecting help-seeking behaviour in Harare, Zimbabwe. Trop Med Int Health.

[59] Boyer S, Clerc I, Bonono CR, Marcellin F, Bilé PC, Ventelou B (2011). Non-adherence to antiretroviral treatment and unplanned treatment interruption among people living with HIV/AIDS in Cameroon: individual and healthcare supply-related factors. Soc Sci Med.

[60] Newman J, Torres P, Azinyue I, Hemingway-Foday J, Atibu J, Akam W (2011). Improvement of service capabilities following the establishment of an electronic database to evaluate AIDS in Central Africa. J Health Inform Dev Ctries.

[61] Brinkhof MW, Dabis F, Myer L, Bangsberg DR, Boulle A, Nash D (2008). Early loss of HIV-infected patients on potent antiretroviral therapy programmes in lower-income countries. Bull World Health Organ.

